# Selenocarbamates As a Prodrug‐Based Approach to Carbonic Anhydrase Inhibition

**DOI:** 10.1002/cmdc.202200085

**Published:** 2022-03-23

**Authors:** Andrea Angeli, Marta Ferraroni, Antonella Capperucci, Damiano Tanini, Gabriele Costantino, Claudiu T. Supuran

**Affiliations:** ^1^ Department NEUROFARBA Sezione di Scienze Farmaceutiche University of Florence Via Ugo Schiff 6 50019 Sesto Fiorentino Florence Italy; ^2^ Department of Food and Drug University of Parma Parco Area delle Scienze, 27/A 43124 Parma Italy; ^3^ Department of Chemistry ‘Ugo Schiff' University of Florence Via Della Lastruccia 3–13 50019 Sesto Fiorentino Firenze Italy

**Keywords:** selenocarbamates, carbonic anhydrase inhibitors, X-ray crystallography, inhibition mechanism, selenium

## Abstract

A study on the activity of selenocarbamates as a novel chemotype acting as carbonic anhydrase (CA, EC 4.2.1.1) inhibitors is reported. Undergoing CA‐mediated hydrolysis, selenocarbamates release selenolates behaving as zinc binding groups and effectively inhibiting CAs. A series of selenocarbamates characterised by high molecular diversity and complexity have been studied against different human CA isoforms such as hCA I, II, IX and XII. Selenocarbamates behave as masked selenols with potential biological applications as prodrugs for CAs inhibition‐based strategies. X‐ray studies provided insights into the binding mode of this novel class of CA inhibitors.

## Graphical abstract

**Selenocarbamates with broad molecular diversity** were synthesised and their inhibitory activities against human carbonic anhydrases (CAs) I, II, IX, and XII were investigated. The CA‐mediated hydrolysis of selenocarbamates enables the release of selenolates, behaving as highly active zinc binding groups. X‐ray co‐crystallographic studies on such potential prodrugs for controlled CA inhibition were also performed.

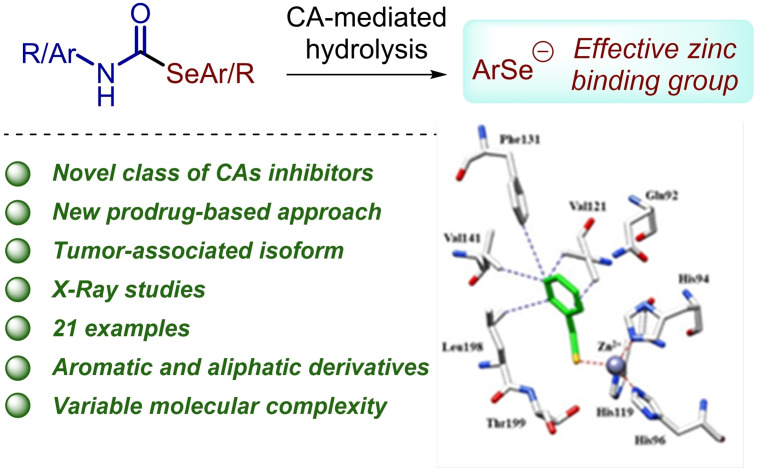

## Introduction

Carbamates and thiocarbamates play an important role in the modern medicinal chemistry for drug and prodrugs synthesis. Owing to their chemical and proteolytic stability, they can also be employed as peptide bond surrogates. Additionally, carbamates and thiocarbamates have been reported to increase permeability across cellular membranes^[1]^ In recent years, these functionalities have also been studied in the field of novel inhibitors of Carbonic Anhydrases (CAs, EC 4.2.1.1).[^2^, ^3^, ^4^] CAs are a family of metalloenzymes wide expressed in all kingdom life involved in a simple anyway crucial reaction, the reversible hydration of CO_2_ into HCO_3_^−^ and H^+^.[^5^, ^6^, ^7^] To date, the most important and largely class used as CA inhibitors (CAIs) are primary sulfonamide derivatives (R‐SO_2_NH_2_).^[8]^ However, the poor selectivity of sulfonamides towards the different CA isoforms represents an important drawback of this excellent zinc binding group (ZBG) which, due to the occurrence of various undesired side‐effects, limits their biomedical employment.^[8]^ As a result, a great emphasis has been placed in recent years on the development of novel ZBGs and prodrugs that, while having lower affinity for the CAs, would allow better modulation of selectivity towards different CA isoforms.[^9^, ^10^, ^11^, ^12^, ^13^]

In this scenario, the unique features of organoselenium compounds are attracting the interest of medicinal chemists. A wide range of biologically active selenium‐containing small molecules have been developed.[^14^, ^15^, ^16^, ^17^, ^18^, ^19^, ^20^, ^21^, ^22^, ^23^, ^24^, ^25^] For example, Ebselen^[26]^ – which represents the most widely studied organoselenium compound – has been very recently demonstrated to possess a remarkable activity against the main protease M^pro^ of SARS‐CoV‐2.^[27]^

Selenium is an essential trace element with important biological functions, involved in the regulation of biochemical pathways having a role on a number of pathologies.^[28]^ Selenium‐containing compounds have emerged as interesting inhibitors of zinc finger proteins.^[29]^ For example, ebselen and selenite have been reported to inhibit cysteine‐rich zinc‐finger transcription factors.^[30]^ The inhibition properties of a range of organoselenium compounds against formamidopyrimidine‐DNA glycosylase^[31]^ and HIV nucleocapsid protein 7 (NCp7)^[32]^ have also been documented.

As a part of our ongoing interest in the study of the biological activity of chalcogen‐containing small molecules,[^33^, ^34^, ^35^] we were interested in evaluating the activity of selenols as carbonic anhydrase inhibitors.[^10^, ^36^] Selenols are better nucleophiles and stronger acids with respect to related thiols (*i. e*. selenocysteine, p*K*_a_=5.8; cysteine, p*K*_a_=8.3). The acidity of selenols enables the rapid generation of nucleophilic selenolates under physiological conditions (pH=7.4). Owing to the facile dissociation of the Se−H bond, the soft character of the selenium atom, and the affinity of selenium compounds for zinc finger proteins, selenols have been recently demonstrated to be a new and potent chemotype acting as human CA inhibitors. However, the relative instability of selenols (which are easily converted to diselenides) significantly hampers their potential application in medicinal chemistry and drug discovery. To overcome the instability of selenols, we considered the possibility of using selenolesters as prodrugs capable of releasing selenols upon CA‐catalysed hydrolysis.^[11]^ Considering the versatility and the biological properties of selenols,^[37]^ the development of new selenol‐based prodrugs is highly desirable. In this context, attracted by the versatility of carbamates in drug discovery and medicinal chemistry[^1^, ^2^, ^38^] we focused our attention on unexplored selenocarbamates.

## Results and Discussion

### Synthesis of selenocarbamates 3

**Scheme 1:**
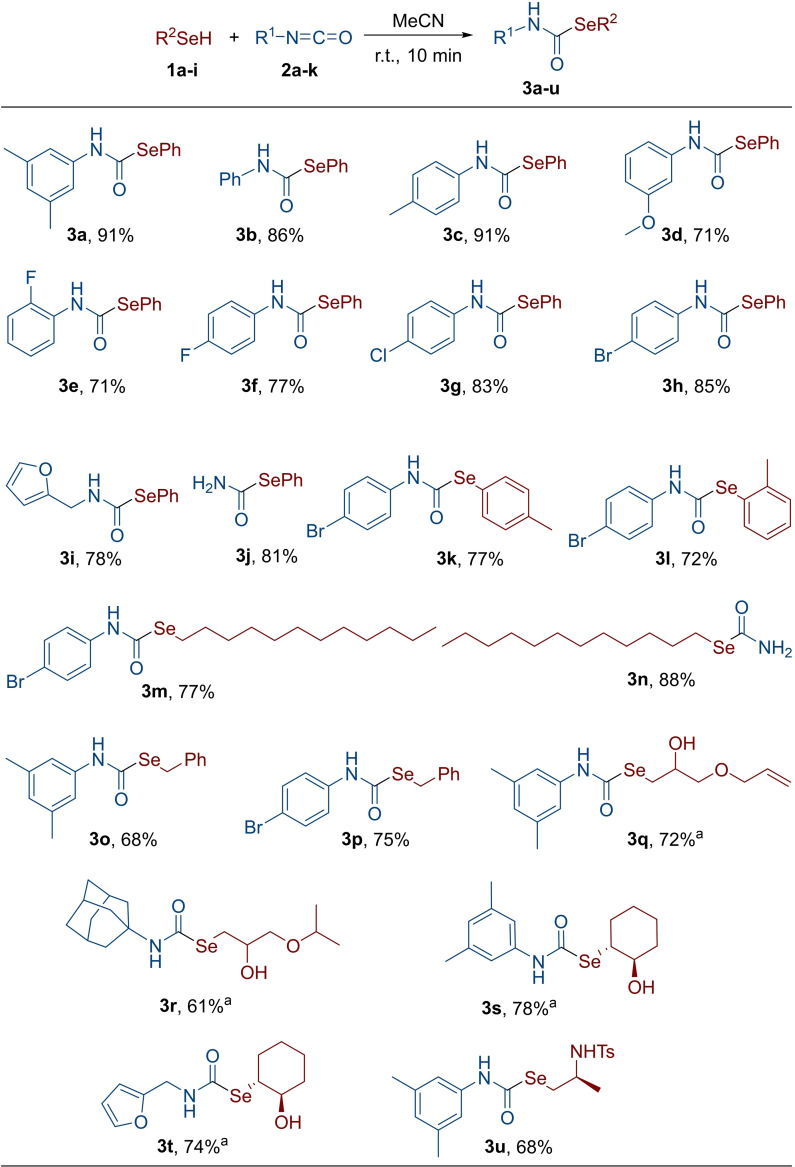
Selenocarbamates used in this work. All yields refer to isolated pure material. Comparable yields were achieved for reactions performed under neat conditions.^[39]^ ^a^ Racemic.

We recently reported that selenocarbamates easily undergo transcarbamoylation reaction providing selenolate anions. Notably, such a reaction was identified as the key step of the unprecedented catalytic cycle accounting for the thiol‐peroxidase‐like activity of selenocarbamates.^[39]^ Variously Se‐ and *N*‐substituted selenocarbamates **3** were prepared from the corresponding selenols and isocyanates by using the catalyst‐free protocol recently developed by some of us.^[39]^ In order to evaluate the effect of the substituent on the N atom, both electron‐rich and electron‐poor *N*‐aryl isocyanates were used in the reaction with benzeneselenol enabling the synthesis of derivatives **3 a**–**d** and **3 e**–**h** (Scheme 1).

The reaction was also applied to the synthesis of the carbamoselenoate **3 i**, bearing the furan moiety. Finally, 1‐(phenylselanyl)formamide **3 j** was achieved from *N*‐trimethylsilyl isocyanate *via* coupling with benzeneselenol and protodesilylation reaction occurring at the carbamoyl nitrogen. To investigate the effect of the substituent on the selenium atom, *p*‐tolyl‐ and *o*‐tolyl selenol were reacted with isocyanate **2 h** enabling the synthesis of selenocarbamates **3 k** and **3 l**. Furthermore, a variety of differently substituted *Se*‐alkyl and *Se*‐benzyl‐carbamoselenoates were obtained. Dodecaneselenol and benzyl selenol were employed for the synthesis of **3 m**–**p**; more complex selenocarbamates **3 q**–**u** were prepared by exploiting the selective reactivity of β‐functionalised alkyl selenols bearing the hydroxy and the amino functionality.

### Carbonic anhydrase inhibition

**Figure 1:**
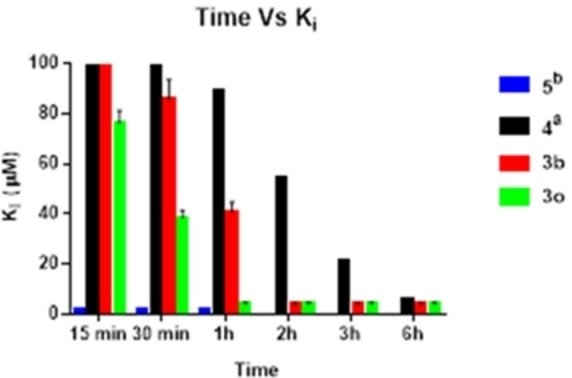
Variation of K_i_ for compounds **3 b**, **3 o**, **4** and **5** versus time. All compounds are incubated with hCA II for 15 min to 6 h h(s). Errors in the range of 10 % of the reported values, from three different stopped‐flow assays.^[40]^ ^a^ See ref. [11]; ^b^ see ref. [10].

**Figure 2:**
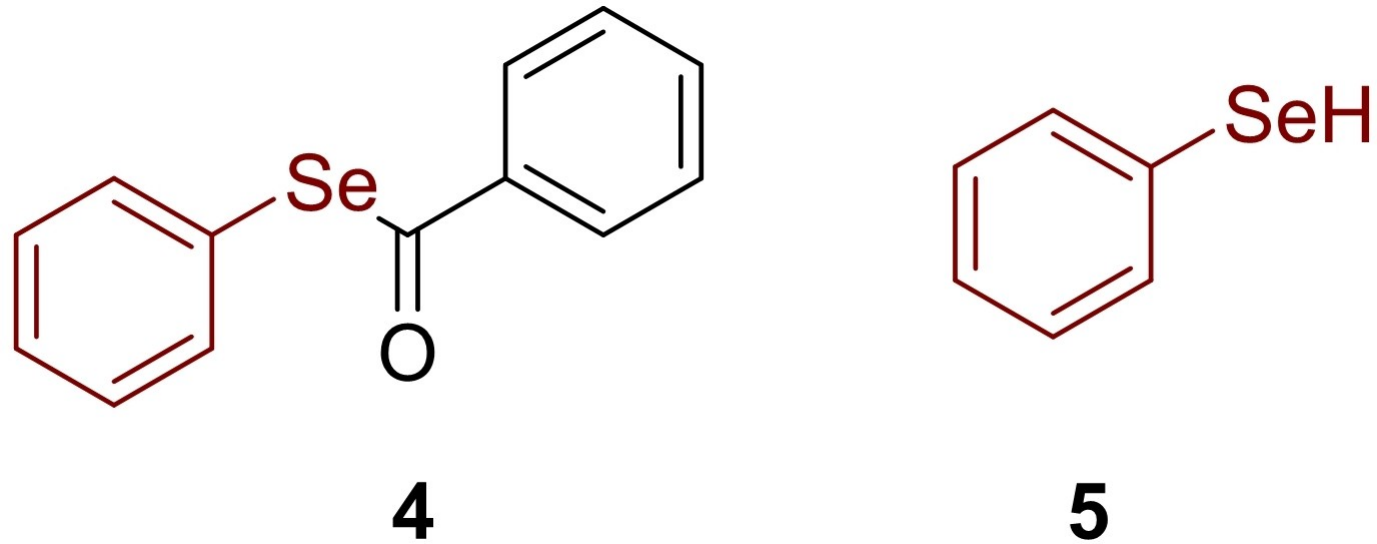
Structure of *Se*‐phenyl benzoselenoate **4** and benzeneselenol **5**.

We began our studies by investigating the stability of selenocarbamates **3 a** and **3 o**, bearing respectively the phenylseleno‐ and the benzylseleno‐moiety, under the same conditions of solvent and temperature used for the kinetic assays. After a time of 12 h, the starting material was recovered without traces of decomposition products (detected by inspection of the ^1^H‐NMR spectra of the raw material, see ESI). Thereafter, the same derivatives were incubated with hCA II and monitored at different time showing the decreased K_i_ values in time‐dependent manner as depicted in Figure 1. These results suggested that compounds **3 b** and **3 o** undergo an enzyme‐promoted chemical transformation similar to what previously reported for selenoesters.^[11]^ In this regard, in order to evaluate the role of the acyl and the carbamoyl moieties, we compared the inhibition profile of selenocarbamates **3 b** and **3 o** with the structurally related *Se*‐phenyl benzoselenoate [**4**, PhSeC(O)Ph] and benzeneselenol (**5**, PhSeH). Structures of compounds **4** and **5** are reported in Figure 2.

From a comparison of inhibition data, when compounds **3 b** and **3 o** were pre‐incubated for 15 minutes, no detectable inhibition was observed for **3 b**, while a high K_i_ value was measured for **3 o**. These data suggested that the benzylseleno motif was more reactive than the aromatic one. In agreement with the previously described hydrolysis reaction of selenoesters, the inhibition constant decreased but, this time, faster than compound **4** reaching the maximum values in 2 h for compound **3 b** and in 1 h for **3 o**. An interesting note regarding the potency of selenolesters and selenocarbamates was the similar K_i_ of **3 b** and **4** with respect to the selenol **5**; these data prove the complete hydrolysis of compounds **3 b** and **4**.

In view of the fact that the selenocarbamate scaffold has proven to be a good CAI, we extended the *in vitro* CA inhibition activities for compounds **3 b**–**u** in comparison to selenolester **4**, benzeneselenol **5** and the clinically sulfonamide acetazolamide (**AAZ**) against four physiologically relevant hCA isozymes such as the ubiquitous hCA I, II and the cancer‐related isoforms hCA IX, XII by means of the stopped‐flow carbon dioxide hydration assay^[21]^ (Table 1).

**Table 1:** Inhibition data of human CA isoforms I, II, IX and XII with compounds **3 a**–**u**, **4**–**5** and **AAZ** by a stopped flow CO_2_ hydrase assay (preincubation time: 6 h).^[40]^

*K*_I_ [μM]^[a]^				
Compd	hCA I	hCA II	hCA IX	hCA XII
**3 a**	49.5	5.3	9.0	8.8
**3 b**	40.4	4.1	47.5	4.6
**3 c**	25.9	8.4	73.4	6.5
**3 d**	49.3	7.9	91.2	7.6
**3 e**	30.8	9.2	66.2	4.4
**3 f**	15.3	9.4	39.5	31.6
**3 g**	42.5	13.1	6.1	8.1
**3 h**	13.8	8.6	9.4	56.8
**3 i**	8.8	40.4	58.1	77.9
**3 j**	9.8	6.8	6.0	7.9
**3 k**	38.6	11.6	26.7	35.7
**3 l**	13.4	8.0	69.2	73.4
**3 m**	92.1	87.4	60.0	53.8
**3 n**	90.3	85.2	67.6	55.7
**3 o**	35.4	4.3	6.7	8.5
**3 p**	85.0	39.9	23.2	35.6
**3 q**	67.5	79.3	>100	>100
**3 r**	92.7	79.4	>100	43.4
**3 s**	74.3	84.6	93.5	56.1
**3 t**	67.4	83.4	>100	46.8
**3 u**	>100	>100	47.7	62.9
**4** ^[11]^	55.7	5.1	26.9	3.9
**5** ^[10]^	6.7	2.2	0.44	n. d.
AAZ	0.25	0.012	0.026	0.006

[a] Data are the mean from three different assays, by a stopped flow technique (errors were in the range of ±5–10 % of the reported values).

From the kinetic data, the nature of the *N*‐aryl substituents did not significantly affect the selectivity, all derivatives showing high potency against hCA II and hCA XII except for compounds with halogen atoms in *para* position **3 f**–**h**. Indeed, while the fluoro‐substituted derivative **3 f** showed selectivity against the ubiquitous hCA I and hCA II, the chloro‐substituted analogue **3 g** displayed selectivity towards tumor‐associated hCA IX and XII. Additionally, the *N*‐4‐bromophenyl‐substituted system **3 h** was found to behave as a selective inhibitors for hCA II and hCA IX. Interestingly, compound **3 j** showed high potency, although with poor selectivity, for all the different CA isoforms, thus proving that the presence of a substituent onto the N atom is essential to modulate the selectivity, particularly against the hCA I. Selenocarbamates with seleno alkyl moiety (**3 m**, **3 n**, **3 q**–**u**) showed low potency of inhibition (high micromolar range) in agreement with our previous studies[^11^, ^36^] on related classes of CA inhibitors. Additionally, these findings are in line with our recent results highlighting that alkylseleno‐substituted selenocarbamates are less prone to undergo transcarbamoylation reactions with respect to their arylseleno‐substituted analogues. Thus, the lower reactivity of the carbamoyl carbon accounting for the poor GPx‐like activity of alkylseleno‐substituted systems reasonably explains also the lower potency against hCAs herein observed.

Another interesting finding is that the 4‐bromo *N*‐aryl moiety proved to considerably decrease the potency of compound **3 p** with respect to **3 o**, which does not contain the 4‐bromo substituent.

### X‐ray studies

**Figure 3:**
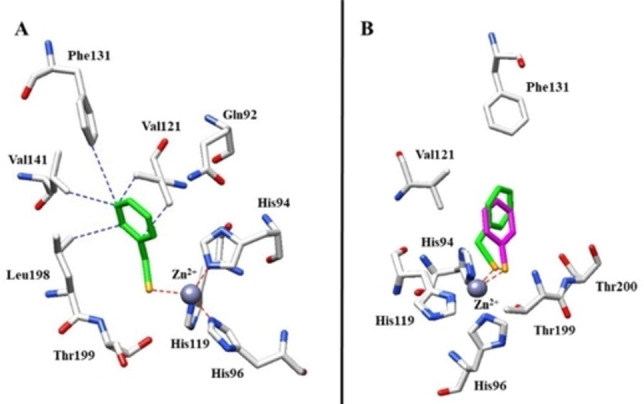
X‐ray crystal structures of hCAII bound with compound **3 o** (A, PDB: 7QBH). Panel B shows the superimposition in the active site among 3o and benzeneselenol **5** (the r.m.s.d. for the superposition is 0.094 Å). Residues involved in the binding of inhibitors are also shown; the grey sphere represents the zinc atom in the active site of the proteins.

To elucidate the binding mode of selenocarbamates to the CA active site, the crystallographic structure of the complex of hCAII with compound **3 o** was determined. Initial rounds of refinement, the Fo−Fc map showed a new density inside the active site of protein, clearly indicating the binding of inhibitor molecule (see Figure S1, in the ESI). This new density map was in agreement with the seleno motif of compound **3 o** and highlights the effective hydrolysis of the selenocarbamate as depicted in Figure 3. The benzylseleno moiety coordinates the zinc ion in a tetrahedral geometry by means of its selenium atom, which replaced the fourth ligand (water molecule/hydroxide ion) present in the free protein. Owing to the low p*K*_a_ of selenols and in line with our previously reported X‐ray studies, benzyl selenol was found in its deprotonated form.[^10^, ^11^, ^36^]

The aromatic ring of the inhibitor is involved in several hydrophobic connections between Val121, Phe131, Val141 and Leu198 (Figure 3A) moving the benzyl moiety towards the hydrophobic region of the active site (Figure S2, ESI) thanks also to the absence of polar interactions between the enzyme and the inhibitor. Interestingly, the structural comparison with benzeneselenol **5** (Figure 3B) showed similar features such as the interaction with the zinc ion by means of a negatively charged selenium atom. On the other hand, the hydrophobic interactions between the protein (Val121, Phe131, Val141 and Leu198) and the benzyl moiety moved the inhibitor in the active site changing the tetrahedral geometry of 16° than benzeneselenol **5** as seen from Figure S3, anyway this difference did not influence the potency of inhibition.

## Conclusion

Taken together, our results clearly indicate that selenocarbamates can act as novel prodrugs against carbonic anhydrase releasing – after CA‐mediated hydrolysis – highly active selenolates which, as confirmed by X‐ray crystallography studies, behave as effective ZBG. Furthermore, differently from previously studied selenolesters and exploiting the reactivity of *in situ* generated carbamic acids, the use of selenocarbamates may also enable the controlled release of amine‐based drugs or bioactive compounds. With these different classes of prodrugs in our arsenal we have the possibility to modulate the inhibition selectivity against the several CA isoforms by the different substituents and the time of hydrolysis in order to modulate also the pharmacokinetic properties.

## Experimental Section

**General**. All commercial materials were purchased from Merck ‐ Sigma‐Aldrich and used as received, without further purification. Solvents were dried using a solvent purification system (Pure‐Solv™). Flash column chromatography purifications were performed with Silica gel 60 (230–400 mesh). Thin layer chromatography was performed with TLC plates Silica gel 60 F254, which was visualised under UV light, or by staining with an ethanolic acid solution of *p*‐anisaldehyde followed by heating. High resolution mass spectra (HRMS) were recorded by Electrospray Ionization (ESI). GC‐MS was performed on a Varian CP 3800/Saturn 2200 instrument. ^1^H and ^13^C NMR spectra were recorded in CDCl_3_ using Varian Mercury 400 and Bruker 400 Ultrashield spectrometers operating at 400 MHz for ^1^H and 100 MHz for ^13^C. ^77^Se NMR spectra were recorded using a Bruker 400 Ultrashield spectrometer, operating at 76 MHz. NMR signals were referenced to nondeuterated residual solvent signals (7.26 ppm for ^1^H, 77.0 ppm for ^13^C). Diphenyl diselenide (PhSe)_2_ was used as an external reference for ^77^Se NMR (*δ*=461 ppm). Chemical shifts (*δ*) are given in parts per million (ppm), and coupling constants (*J*) are given in Hertz (Hz), rounded to the nearest 0.1 Hz. ^1^H NMR data are reported as follows: chemical shift, integration, multiplicity (s=singlet, d=doublet, t=triplet, ap d=apparent doublet, m=multiplet, dd=doublet of doublet, bs=broad singlet, bd=broad doublet, etc.), coupling constant (*J*) or line separation (ls), and assignment. Where reported, NMR assignments are made according to spin systems, using, where appropriate, 2D NMR experiments (COSY, HSQC, HMBC) to assist the assignment.

β‐Hydroxy‐ and β‐amino‐selenols were synthesised from the corresponding epoxides and aziridines following a reported procedure.^[41]^ Aryl‐selenols, dodecane‐1‐selenol, and phenylmethaneselenol were prepared through a reported procedure from the corresponding diselenides upon reduction with NaBH_4_ followed by treatment with citric acid.^[10]^

**General procedure for the synthesis of selenocarbamates 3**. Selenocarbamates were prepared following our recently developed procedure.^[39]^ To a stirred solution of selenol **1** (1.0 mmol, 1.1 equiv.) in anhydrous acetonitrile (1 mL) at room temperature under a nitrogen atmosphere, isocyanate **2** (0.91 mmol, 1.0 equiv.) was added. After stirring for 10 minutes the solvent was removed under vacuum and the crude material purified by precipitation or subjected to flash column chromatography (petroleum ether/Ethyl acetate) to afford selenocarbamates **3**.

**Carbonic anhydrase inhibition**. An Applied Photophysics stopped‐flow instrument was used to assay the CA catalyzed CO_2_ hydration activity.^[40]^ Phenol red (at a concentration of 0.2 mM) was used as an indicator, working at the absorbance maximum of 557 nm, with 20 mM HEPES (pH 7.4) as a buffer, and 20 mM Na_2_SO_4_ (to maintain constant ionic strength), following the initial rates of the CA‐catalyzed CO_2_ hydration reaction for a period of 10–100 s. The CO_2_ concentrations ranged from 1.7 to 17 mM for the determination of the kinetic parameters and inhibition constants.^[6]^ Enzyme concentrations ranged between 5–12 nM. For each inhibitor, at least six traces of the initial 5–10 % of the reaction were used to determine the initial velocity. The uncatalyzed rates were determined in the same manner and subtracted from the total observed rates. Stock solutions of the inhibitor (0.1 mM) were prepared in distilled–deionized water and dilutions up to 0.01 nM were done thereafter with the assay buffer. Inhibitor and enzyme solutions were preincubated together for 15 min to 6 h at room temperature prior to the assay, to allow for the formation of the E–I complex. The inhibition constants were obtained by non‐linear least‐squares methods using PRISM 3 and the Cheng‐Prusoff equation as reported earlier and represent the mean from at least three different determinations. All CA isoforms were recombinant proteins obtained in house, as reported earlier.[^42^, ^43^]

**Crystallization and X‐ray data collection**. Crystals of hCAII were obtained using the hanging drop vapor diffusion method using 24 well Linbro plate. 2 μl of 10 mg/ml solution of hCA II in Tris‐HCl 20 mM pH 8.0 were mixed with 2 μl of a solution of 1.5 M sodium citrate, 0.1 M Tris pH 8.0 and were equilibrated against the same solution at 296 K. The complexes were prepared by soaking the hCA II native crystals in the mother liquor solution containing the inhibitors at concentration of 10 mM for 6 h. All crystals were flash‐frozen at 100 K using a solution obtained by adding 15 % (v/v) glycerol to the mother liquor solution as cryoprotectant. Data on crystal of the complex was collected using synchrotron radiation at the XRD2 beamline at Elettra Synchrotron (Trieste, Italy) with a wavelength of 0.971700 Å and a DECTRIS Pilatus 6 M detector. Data were integrated and scaled using the program XDS.^[44]^

Full experimental details are reported in the Supporting Information.

## Conflict of interest

The authors declare no conflict of interest.

1

## Supporting information

As a service to our authors and readers, this journal provides supporting information supplied by the authors. Such materials are peer reviewed and may be re‐organized for online delivery, but are not copy‐edited or typeset. Technical support issues arising from supporting information (other than missing files) should be addressed to the authors.

Supporting Information

## Data Availability

The data that support the findings of this study are available from the corresponding author upon reasonable request.
