# Supplementary material for: Selenocarbamates As a Prodrug‐Based Approach to Carbonic Anhydrase Inhibition
Source: ChemMedChem. 2022 Mar 23;17(11):e202200085. doi: 10.1002/cmdc.202200085 (PMC9310613; doi:10.1002/cmdc.202200085)
Supplement: Supplementary file 1 — Supporting Information [file CMDC-17-0-s001.pdf]

ChemMedChem

Supporting Information

Selenocarbamates As a Prodrug-Based Approach to Carbonic Anhydrase Inhibition

Andrea Angeli, Marta Ferraroni, Antonella Capperucci, Damiano Tanini,*
Gabriele Costantino, and Claudiu T. Supuran

Contents

1. Synthesis and characterisation of selenocarbamates **3** *S1*

2. ^{1}H-NMR spectra of control experiments for compounds **3a** and **3o** *S8*

2. Structure determination *S10*

3. Summary of Data Collection and Atomic Model Refinement Statistics for hCA II *S11*

4. Crystallographic figures **S2-S4** *S12*

5. References *S13*

1. Synthesis and characterisation of selenocarbamates 3

General procedure for the synthesis of selenocarbamates 3

Selenocarbamates were prepared following our recently developed procedure.[1] To a stirred solution of selenol **1** (1.0 mmol, 1.1 equiv.) in anhydrous acetonitrile (1 mL) at room temperature under a nitrogen atmosphere, isocyanate **2** (0.91 mmol, 1.0 equiv.) was added. After stirring for 10 minutes the solvent was removed under vacuum and the crude material purified by precipitation or subjected to flash column chromatography (petroleum ether/Ethyl acetate) to afford selenocarbamates **3**.

Synthesis of *Se*-phenyl (3,5-dimethylphenyl)carbamoselenoate 3a

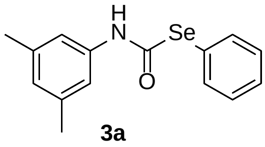

3a

Following the General Procedure, benzeneselenol **1a** (126 mg, 0.8 mmol) and 1-isocyanato-3,5-dimethylbenzene **2a** (107 mg, 0.73 mmol) gave, after precipitation from Et_2O/pentane, **3a** as a white solid (201 mg, 91%). **^{1}H NMR** ($CDCl_3$, 400 MHz) δ (ppm): 2.25 (6H, s), 6.75 (1H, s), 6.93 (2H, s), 7.01 (1H, bs, NH), 7.42-7.48 (3H, m), 7.73-7.75 (2H, m). **^{13}C NMR** ($CDCl_3$, 100 MHz) δ (ppm): 21.9, 117.8 (bs), 127.2 (bs), 130.3, 130.5, 137.3, 137.8, 139.5. **^{77}Se NMR** ($CDCl_3$, 76 MHz) δ (ppm): 538.3.

Synthesis of *Se*-phenyl phenylcarbamoselenoate 3b

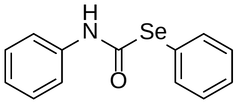

3b

Following the General Procedure, benzeneselenol **1a** (63 mg, 0.4 mmol) and isocyanatobenzene **2b** (44 mg, 0.37 mmol) gave, after precipitation from Et_2O/pentane, **3b** as a white solid (87 mg, 86%). **^{1}H NMR** ($CDCl_3$, 400 MHz) δ (ppm): 7.16 (1H, ap t, J = 6.8 Hz), 7.31-7.38 (5H, m), 7.46-7.53 (3H, m), 7.79-7.81 (2H, m). **^{13}C NMR** ($CDCl_3$, 100 MHz) δ (ppm): 119.4 (bs), 124.7, 126.6, 129.0, 129.7, 129.8, 136.6, 137.3, 161.2 (bs).

Synthesis of *Se*-phenyl *p*-tolylcarbamoselenoate 3c

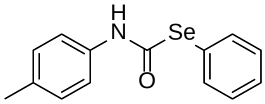

3c

Following the General Procedure, benzeneselenol **1a** (63 mg, 0.4 mmol) and 1-isocyanato-4-methylbenzene **2c** (49 mg, 0.37 mmol) gave, after precipitation from Et_2O/pentane, **3c** as a

white solid (96 mg, 91%). **^{1}H NMR** ($CDCl_3$, 400 MHz) δ (ppm): 2.30 (3H, s), 7.08 (2H, ap d, J = 8.2 Hz), 7.14 (1H, bs, NH), 7.20 (2H, ap d, J = 8.2 Hz), 7.41-7.48 (3H, m), 7.75 (2H, ap d, J = 6.6 Hz). **^{13}C NMR** ($CDCl_3$, 100 MHz) δ (ppm): 20.8, 119.2 (bs), 126.7, 129.56, 129.61, 129.8, 134.8, 136.6.

Synthesis of *Se*-phenyl (3-methoxyphenyl)carbamoselenoate 3d

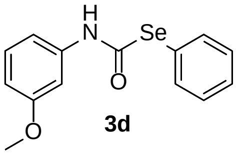

3d

Following the General Procedure A, benzeneselenol **1a** (94 mg, 0.6 mmol) and 1-isocyanato-3-methoxybenzene **2d** (81 mg, 0.55 mmol) gave, after precipitation from Et_2O/pentane, **3d** as a pale yellowish solid (120 mg, 71%). **^{1}H NMR** ($CDCl_3$, 400 MHz) δ (ppm): 3.80 (3H, s), 6.71 (1H, dd, J = 1.8, 8.1 Hz), 6.81 (1H, d, J = 8.1 Hz), 7.12 (1H, t, J = 1.8 Hz), 7.21 (1H, t, J = 8.1 Hz), 7.32 (1H, bs, NH), 7.45-7.52 (3H, m), 7.77-7.79 (2H, m). **^{13}C NMR** ($CDCl_3$, 100 MHz) δ (ppm): 55.3, 105.2 (bs), 110.6, 111.5 (bs), 129.8, 129.9, 136.7, 138.5, 160.2, 161.3 (bs). **^{77}Se NMR** ($CDCl_3$, 76 MHz) δ (ppm): 541.4.

Synthesis of *Se*-phenyl (2-fluorophenyl)carbamoselenoate 3e

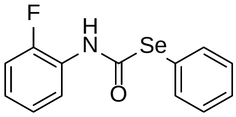

3e

Following the General Procedure A, benzeneselenol **1a** (63 mg, 0.4 mmol) and 1-fluoro-2-isocyanatobenzene **2e** (50 mg, 0.37 mmol) gave, after precipitation from Et_2O/pentane, **3e** as a pale yellowish solid (76 mg, 71%). **^{1}H NMR** ($CDCl_3$, 400 MHz) δ (ppm): 6.99-7.16 (4H, m), 7.36 (1H, bs, NH), 7.44-7.52 (3H, m), 7.77-7.78 (1H, m), 8.12 (1H, t, J = 7.8 Hz). **^{13}C NMR** ($CDCl_3$, 100 MHz) δ (ppm): 114.9 (d, $^2J_{C\text{-}F}$ = 18.9 Hz), 121.1 (bs), 121.7 (bs), 124.6, 124.7, 124.8, 126.2, 130.0, 161.5 (bs). **^{77}Se NMR** ($CDCl_3$, 76 MHz) δ (ppm): 544.3.

Synthesis of *Se*-phenyl (4-fluorophenyl)carbamoselenoate 3f

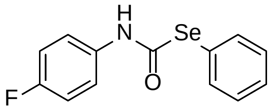

3f

Following the General Procedure A, benzeneselenol **1a** (126 mg, 0.8 mmol) and 1-fluoro-4-isocyanatobenzene **2f** (100 mg, 0.73 mmol) gave, after precipitation from Et_2O/pentane, **3f** as a pale yellowish solid (165 mg, 77%). **^{1}H NMR** ($CDCl_3$, 400 MHz) δ (ppm): 7.02 (2H, t, J = 8.6 Hz), 7.19 (1H, bs, NH), 7.30-7.34 (2H, m), 7.46-7.54 (3H, m), 7.78-7.80 (2H, m). **^{13}C NMR** ($CDCl_3$, 100 MHz) δ (ppm): 116.4 (d,

$^{2}J_{C-F}$ = 22.7 Hz), 121.9 (bs), 127.1, 130.4, 130.5, 134.0, 137.3, 160.3 (bd, $^{1}J_{C-F}$ = 246.2 Hz). **^{77}Se NMR** ($CDCl_3$, 76 MHz) δ (ppm): 537.6.

Synthesis of *Se*-phenyl (4-chlorophenyl)carbamoselenoate 3g

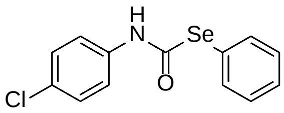

3g

Following the General Procedure A, benzeneselenol **1a** (63 mg, 0.4 mmol) and 1-chloro-4-isocyanatobenzene **2g** (56 mg, 0.37 mmol) gave, after precipitation from Et_2O/pentane, **3g** as a colourless solid (94 mg, 83%). **^{1}H NMR** ($CDCl_3$, 400 MHz) δ (ppm): 7.21-7.26 (5H, m), 7.41-7.48 (3H, m), 7.72-7.74 (2H, m). **^{13}C NMR** ($CDCl_3$, 100 MHz) δ (ppm): 120.7 (bs), 126.3, 129.1, 129.8, 129.9, 135.9, 136.7, 161.6 (bs).

Synthesis of *Se*-phenyl (4-bromophenyl)carbamoselenoate 3h

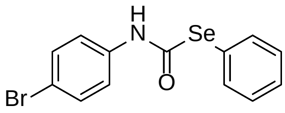

3h

Following the General Procedure A, benzeneselenol **1a** (94 mg, 0.6 mmol) and 1-bromo-4-isocyanatobenzene **2h** (109 mg, 0.55 mmol) gave, after precipitation from Et_2O/pentane, **3h** as a white solid (166 mg, 85%). **^{1}H NMR** ($CDCl_3$, 400 MHz) δ (ppm): 7.21 (2H, d, J = 8.7 Hz), 7.37 (2H, d, J = 8.7 Hz), 7.41-7.48 (4H, m overlapped with bs of NH), 7.73 (2H, ap d, ls = 6.5 Hz). **^{13}C NMR** ($CDCl_3$, 100 MHz) δ (ppm): 118.0 (bs), 121.7 (bs), 127.0, 130.4, 130.5, 132.7, 137.1, 137.3, 162.4 (bs).

Synthesis of *Se*-phenyl (furan-2-ylmethyl)carbamoselenoate 3i

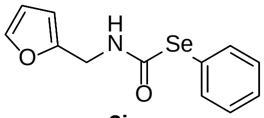

3i

Following the General Procedure A, benzeneselenol **1a** (86 mg, 0.5 mmol) and 2-(isocyanatomethyl)furan **2i** (55 mg, 0.45 mmol) gave, after precipitation from Et_2O/pentane, **3i** as a brownish oil (98 mg, 81%). **^{1}H NMR** ($CDCl_3$, 400 MHz) δ (ppm): 4.40 (2H, d, J = 5.5 Hz), 5.83 (1H, bs), 6. 18 (1H, d, J = 2.6 Hz), 6.29-6.30 (1H, m), 7.32 (1H, ap s), 7.33-7.41 (3H, m), 7.66-7.68 (2H, m). **^{13}C NMR** ($CDCl_3$, 100 MHz) δ (ppm): 38.3, 107.7, 110.4, 126.3, 129.1, 129.4, 129.6, 131.5, 136.5, 142.3, 150.3, 162.8.

Synthesis of *Se*-phenyl carbamoselenoate 3j

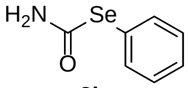

3j Following the General Procedure A, benzeneselenol **1a** (63 mg, 0.4 mmol) and isocyanatotrimethylsilane **2j** (42 mg, 0.37 mmol) gave, after precipitation from Et_2O/hexane, **3j** as a white solid (59 mg, 78%). ^{1}**H NMR** ($CDCl_3$, 400 MHz) δ (ppm): 5.70 (2H, bs, NH_2), 7.39-7.44 (3H, m), 7.69-7.62 (2H, m). ^{13}**C NMR** ($CDCl_3$, 100 MHz) δ (ppm): 126.7 (C), 129.7 (CH), 129.8 (CH), 136.6 (CH), 165.7 (C).

Synthesis of *Se*-(*p*-tolyl) (4-bromophenyl)carbamoselenoate 3k

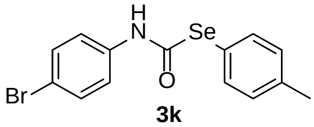

3k Following the General Procedure A, 4-methylbenzeneselenol **1b** (86 mg, 0.5 mmol) and 1-bromo-4-isocyanatobenzene **2h** (90 mg, 0.45 mmol) gave, after precipitation from Et_2O/pentane, **3k** as a yellowish solid (127 mg, 77%). ^{1}**H NMR** ($CDCl_3$, 400 MHz) δ (ppm): 2.41 (3H, s), 7.08 (1H, bs, NH), 7.19 (2H, d, *J* = 8.1 Hz), 7.24-7.26 (2H, m), 7.38 (2H, d, *J* = 8.1 Hz), 7.62 (2H, d, *J* = 7.7 Hz). ^{13}**C NMR** ($CDCl_3$, 100 MHz) δ (ppm): 21.4, 120.8 (bs), 122.8, 130.9, 132.0, 136.4, 136.7, 140.4.

Synthesis of *Se*-(*o*-tolyl) (4-bromophenyl)carbamoselenoate 3l

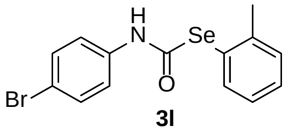

3l Following the General Procedure A, 2-methylbenzeneselenol **1c** (43 mg, 0.25 mmol) and 1-bromo-4-isocyanatobenzene **2h** (45 mg, 0.23 mmol) gave, after precipitation from Et_2O/pentane, **3l** as a yellowish solid (59 mg, 72%). ^{1}**H NMR** ($CDCl_3$, 400 MHz) δ (ppm): 2.55 (3H, s), 7.03 (1H, bs, NH), 7.15-7.19 (2H, m), 7.23-7.27 (1H, m), 7.36-7.39 (2H, m), 7.40-7.41 (2H, m), 7.77 (1H, ap d, *J* = 7.5 Hz). ^{13}**C NMR** ($CDCl_3$, 100 MHz) δ (ppm): 23.4, 120.8, 127.4, 127.6, 130.8, 131.0, 132.1, 136.4, 138.3, 142.9.

Synthesis of *Se*-dodecyl (4-bromophenyl)carbamoselenoate 3m

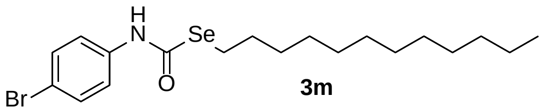

3m Following the General Procedure A, dodecane-1-selenol **1d** (63 mg, 0.25 mmol) and 1-bromo-4-isocyanatobenzene **2h** (45 mg, 0.23 mmol) gave, after precipitation from Et_2O/cyclohexane, **3m** as a white solid (78 mg, 77%). ^{1}**H NMR** ($CDCl_3$, 400 MHz) δ (ppm): 0.86 (3H, t, *J*

= 6.7 Hz), 1.23-1.31 (16H, m), 1.32-1.41 (2H, m), 1.69-1.76 (2H, m), 2.99 (2H, t, *J* = 7.3 Hz, CH_2Se), 7.24 (1H, bs, NH), 7.29 (2H, ap d, *J* = 8.6 Hz), 7.39 (2H, ap d, *J* = 8.6 Hz). **^{13}C NMR** ($CDCl_3$, 100 MHz) δ (ppm): 14.1, 22.7, 27.7, 29.1, 29.3, 29.5, 29.57, 29.61, 29.63, 29.9, 30.9, 31.9, 120.3, 121.2, 132.1, 136.7, 162.0.

Synthesis of *Se*-dodecyl carbamoselenoate 3n

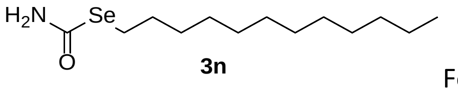

3n

Following the General Procedure A, dodecane-1-selenol **1d** (63 mg, 0.25 mmol) and isocyanatotrimethylsilane **2j** (26 mg, 0.23 mmol) gave, after precipitation from Et_2O/hexane, **3n** as a white solid (58 mg, 88%). **^{1}H NMR** ($CDCl_3$, 400 MHz) δ (ppm): 0.88 (3H, t, *J* = 6.8 Hz), 1.21-1.33 (18H, m), 1.66-1.75 (2H, m), 2.95 (2H, t, *J* = 7.3 Hz, CH_2Se), 5.56 (2H, bs, NH_2). **^{13}C NMR** ($CDCl_3$, 100 MHz) δ (ppm): 14.1, 22.7, 27.3, 29.1, 29.4, 29.5, 29.6, 29.7, 29.9, 30.9, 31.9, 165.2.

Synthesis of *Se*-benzyl (3,5-dimethylphenyl)carbamoselenoate 3o

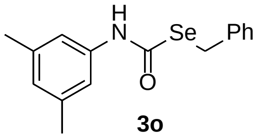

3o

Following the General Procedure A, phenylmethaneselenol **1e** (103 mg, 0.6 mmol) and 1-isocyanato-3,5-dimethylbenzene **2a** (81 mg, 0.55 mmol) gave, after precipitation from Et_2O/cyclohexane, **3o** as a pale yellowish solid (118 mg, 67%). **^{1}H NMR** ($CDCl_3$, 400 MHz) δ (ppm): 2.29 (6H, s), 4.26 (2H, s, CH_2Se), 6.79, (1H, s), 6.97 (1H, s), 7.03 (2H, s), 7.20-7.23 (1H, m), 7.27-7.31 (2H, m), 7.35-7.37 (2H, m). **^{13}C NMR** ($CDCl_3$, 100 MHz) δ (ppm): 21.9, 31.2, 127.7, 129.3, 129.6, 139.6, 139.8, 153.0, 160.4 (C=O).

Synthesis of *Se*-benzyl (4-bromophenyl)carbamoselenoate 3p

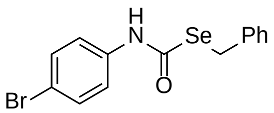

3p

Following the General Procedure A, phenylmethaneselenol **1e** (52 mg, 0.3 mmol) and 1-bromo-4-isocyanatobenzene **2h** (55 mg, 0.28 mmol) gave, after precipitation from Et_2O/cyclohexane, **3p** as a pale yellowish solid (65 mg, 75%). **^{1}H NMR** ($CDCl_3$, 400 MHz) δ (ppm): 4.27 (2H. s, CH_2Se), 7.13 (1H, bs, NH), 7.21-7.36 (7H, m), 7.42-7.44 (2H, m). **^{13}C NMR** ($CDCl_3$, 100 MHz) δ (ppm): 31.4, 121.9 (bs), 127.8, 129.3, 129.6, 132.8, 137.2 (bs), 139.5, 162.4 (bs). **MS** (ESI): 391.8 $[M+Na]^+$.

Synthesis of *Se*-(2-(allyloxy)-2-hydroxyethyl) (3,5-dimethylphenyl)carbamoselenoate 3q

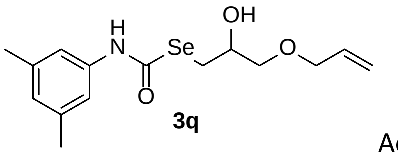

3q

According to the General Procedure A, the reaction was carried out with 1-(allyloxy)-3-hydroselenopropan-2-ol **1f** (69 mg, 0.35 mmol) and 1-isocyanato-3,5-dimethylbenzene **2a** (47 mg, 0.32 mmol). Purification by flash column chromatography (petroleum ether/EtOAc 4:1) gave **3q** as a colourless oil (78 mg, 72%). ^{1}**H NMR** ($CDCl_3$, 400 MHz) δ (ppm): 2.29 (6H, s), 2.91 (1H, bs, OH), 3.11 (1H, dd, *J* = 6.9, 13.4 Hz, C$\mathbf{H_a}$H$_b$Se), 3.24 (1H, dd, *J* = 4.1, 13.4 Hz, CH$_a$$\mathbf{H_b}$Se), 3.49 (1H, dd, *J* = 6.7, 9.6 Hz, C$\mathbf{H_a}$H$_b$O), 3.55 (1H, dd, *J* = 4.4, 9.6 Hz, CH$_a$$\mathbf{H_b}$O), 4.03-4.04 (2H, m, OC$\mathbf{H_2}$CH=CH$_2$), 4.06-4.13 (1H, m, C**H**OH), 5.19-5.30 (2H, m, CH=C$\mathbf{H_2}$), 5.86-5.95 (1H, m, C**H**=CH$_2$), 6.79 (1H, s), 7.03 (2H, s), 7.38 (1H, bs, NH). ^{13}**C NMR** ($CDCl_3$, 100 MHz) δ (ppm): 21.3, 30.5, 70.3, 72.3, 73.2, 117.4, 126.5, 134.3, 138.9.

Synthesis of *Se*-(2-hydroxy-3-isopropoxypropyl) (adamantan-1-yl)carbamoselenoate 3r

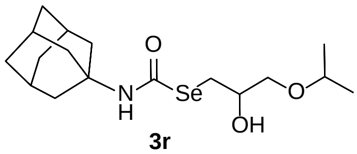

3r

According to the General Procedure A, the reaction was carried out with 1-hydroseleno-3-isopropoxypropan-2-ol **1g** (50 mg, 0.25 mmol) and 1-adamantyl isocyanate **2k** (40 mg, 0.23 mmol). Purification by flash column chromatography (petroleum ether/EtOAc 3:1) gave **3r** as a colourless oil (51 mg, 61%). ^{1}**H NMR** ($CDCl_3$, 400 MHz) δ (ppm): 1.14 (3H, d, *J* = 6.1 Hz), 1.15 (3H, d, *J* = 6.1 Hz), 1.65-1.66 (6H, m), 1.96-1.97 (6H, m), 2.05-2.10 (3H, m), 2.60 (1H, bs, OH), 2.99 (1H, dd, *J* = 6.9, 13.3 Hz, C$\mathbf{H_a}$H$_b$Se), 3.11 (1H, dd, *J* = 4.2, 13.3 Hz, CH$_a$$\mathbf{H_b}$Se), 3.40 (1H, dd, *J* = 6.4, 9.4 Hz, C$\mathbf{H_a}$H$_b$O), 3.47 (1H, dd, *J* = 4.8, 9.4, CH$_a$$\mathbf{H_b}$O), 3.54-3.64 (1H, m, C**H**(CH$_3$)$_2$), 3.92-3.98 (1H, m, C**H**OH), 5.34 (1H, bs, NH). ^{13}**C NMR** ($CDCl_3$, 100 MHz) δ (ppm): 22.1, 29.4, 30.5, 36.1, 41.8, 54.9, 70.4, 71.2, 72.2, 160.7. **MS** (ESI): 376.3 $[M+H]^+$.

Synthesis of *Se*-(2-hydroxycyclohexyl) (3,5-dimethylphenyl)carbamoselenoate 3s

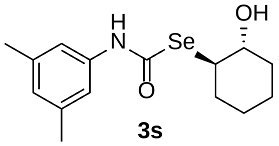

3s

According to the General Procedure A, the reaction was carried out with 2-hydroselenocyclohexan-1-ol **1h** (180 mg, 1 mmol) and 1-isocyanato-3,5-dimethylbenzene **2a** (134 mg, 0.91 mmol). Purification by flash column chromatography (petroleum ether/EtOAc 4:1) gave **3s** as a colourless oil (232 mg, 78%). ^{1}**H NMR** ($CDCl_3$, 400 MHz) δ (ppm): 1.24-1.41 (4H, m), 1.54-1.64 (1H, m), 1.65-1.79 (2H, m), 2.11-2.22 (1H, m), 2.27 (6H, s), 3.00 (1H, bs, OH), 3.38 (1H, ddd, *J* = 4.0, 10.1, 12.4 Hz, CHSe), 3.60 (1H,

td, *J* = 4.1, 10.1 Hz), 6.76 (1H, s), 7.03 (2H, s), 7.86 (1H, bs, NH). **^{13}C NMR** ($CDCl_3$, 100 MHz) δ (ppm): 21.3, 24.3, 26.8, 32.8, 35.7, 51.9, 74.7, 117.4, 126.5, 138.9.

Synthesis of *Se*-2-hydroxycyclohexyl) (furan-2-ylmethyl)carbamoselenoate 3t

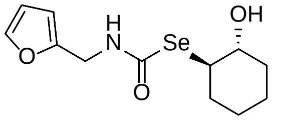

3t

According to the General Procedure A, the reaction was carried out with 2-hydroselenocyclohexan-1-ol **1h** (180 mg, 1 mmol) and 2-(isocyanatomethyl)furan **2i** (112 mg, 0.91 mmol). Purification by flash column chromatography (petroleum ether/EtOAc 3:1) gave **3t** as a brownish oil (203 mg, 74%). **^{1}H NMR** ($CDCl_3$, 400 MHz) δ (ppm): 1.31-1.39 (3H, m), 1.51-1.60 (1H, m), 1.65-1.66 (1H, m), 1.76-1.77 (1H, m), 2.11-2.14 (1H, m), 2.19-2.26 (1H, m), 2.85 (1H, bs), 3.30-3.41 (1H, m), 3.50-3.55 (1H, m), 4.40 (1H, dd, *J* = 5.3, 15.4 Hz), 4.46 (1H, dd, *J* = 5.4, 15.4 Hz), 6.23-6.24 (1H, m), 6.30-6.31 (2H, m overlapped with bs), 7.34 (1H, ap s). **^{13}C NMR** ($CDCl_3$, 100 MHz) δ (ppm): 24.3, 26.8, 32.8, 35.6, 38.4, 51.6, 74.6, 108.0, 110.5, 142.5, 150.4, 164.1.

Synthesis of (*S*)-*Se*-(2-((4-methylphenyl)sulfonamido)propyl) (3,5-dimethylphenyl)carbamoselenoate 3u

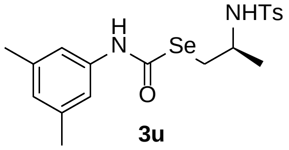

3u

According to the General Procedure A, the reaction was carried out with (*S*)-*N*-(1-hydroselenopropan-2-yl)-4-methylbenzenesulfonamide **1i** (59 mg, 0.2 mmol) and 1-isocyanato-3,5-dimethylbenzene **2a** (27 mg, 0.18 mmol). Purification by flash column chromatography (petroleum ether/EtOAc 3:1) gave **3u** as a white powder (54 mg, 68%). **^{1}H NMR** (Acetone-d_6, 400 MHz) δ (ppm): 1.16 (3H, d, *J* = 6.5 Hz), 2.26 (6H, s), 2.37 (3H, s), 3.00-3.05 (2H, m, CH_2Se), 3.49-3.58 (1H, m, C**H**N), 6.45 (1H, d, *J* = 6.9 Hz, N**H**Ts), 6.76 (1H, s), 7.18 (2H, s), 7.35 (2H, ap d, *J* = 8.5 Hz), 7.76 (2H, ap d, *J* = 8.5 Hz), 9.22 (1H, s, N**H**C(O)). **^{13}C NMR** (Acetone-d_6, 100 MHz) δ (ppm): 20.4, 20.5, 20.7, 32.5, 50.4, 116.8, 125.4, 126.9, 129.4, 138.4, 138.7, 139.0, 142.8, 160.4 (C=O).

2. ^{1}H-NMR spectra of control experiments for compounds **3a** and **3o**

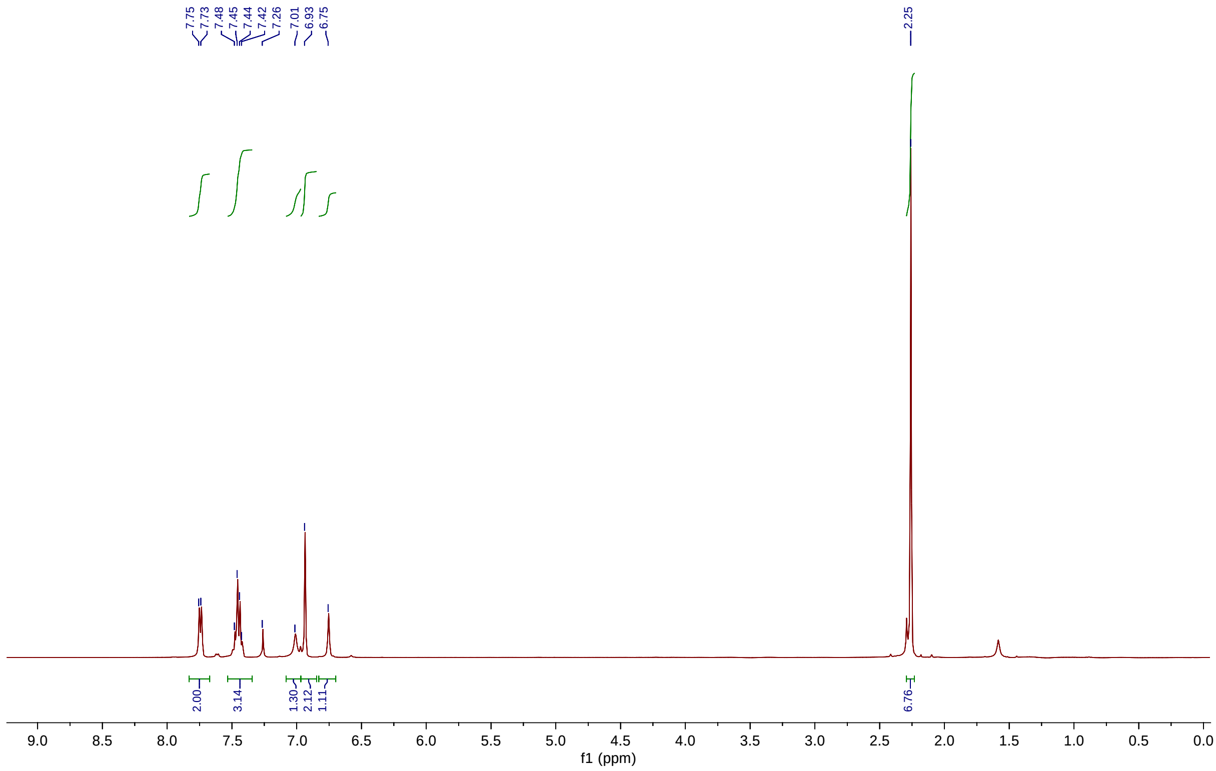

^{1}H NMR Spectrum of compound **3a** ($CDCl_3$, 400 MHz)

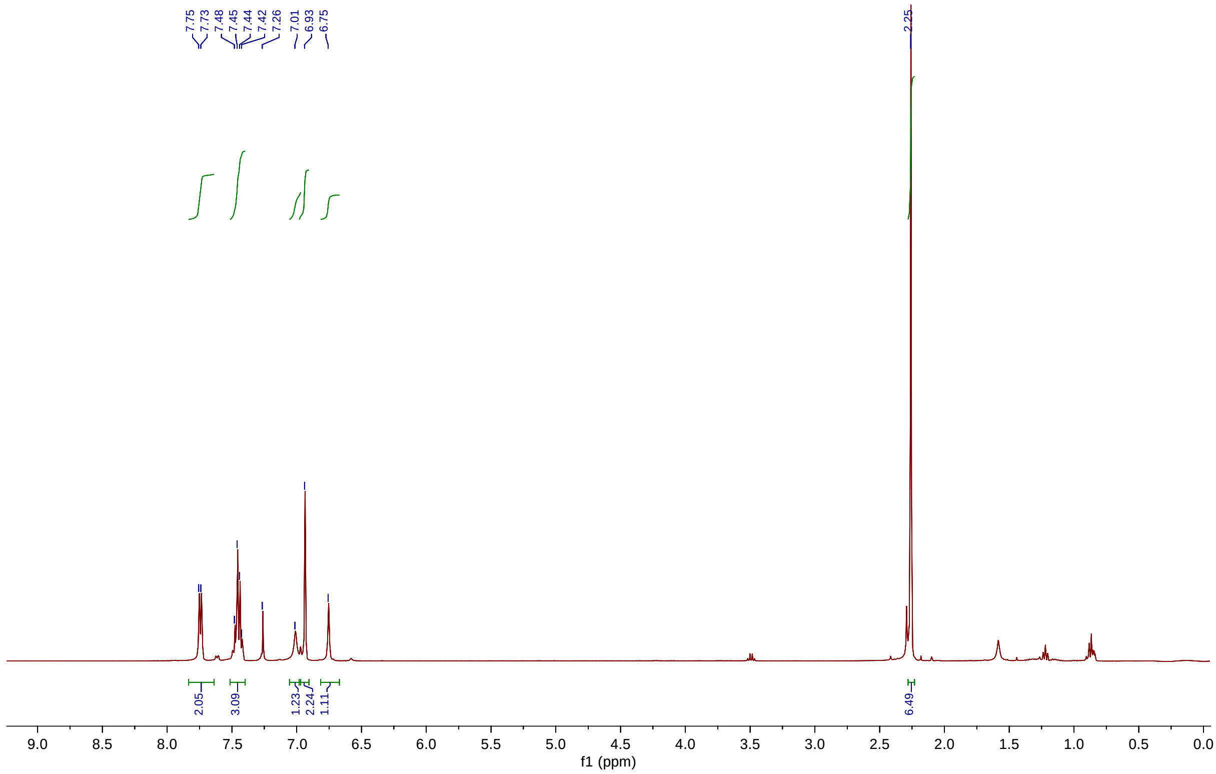

^{1}H NMR spectrum of the raw material recovered after stirring the selenocarbamate **3a** in the buffer solution and under conditions used for the kinetic assays.

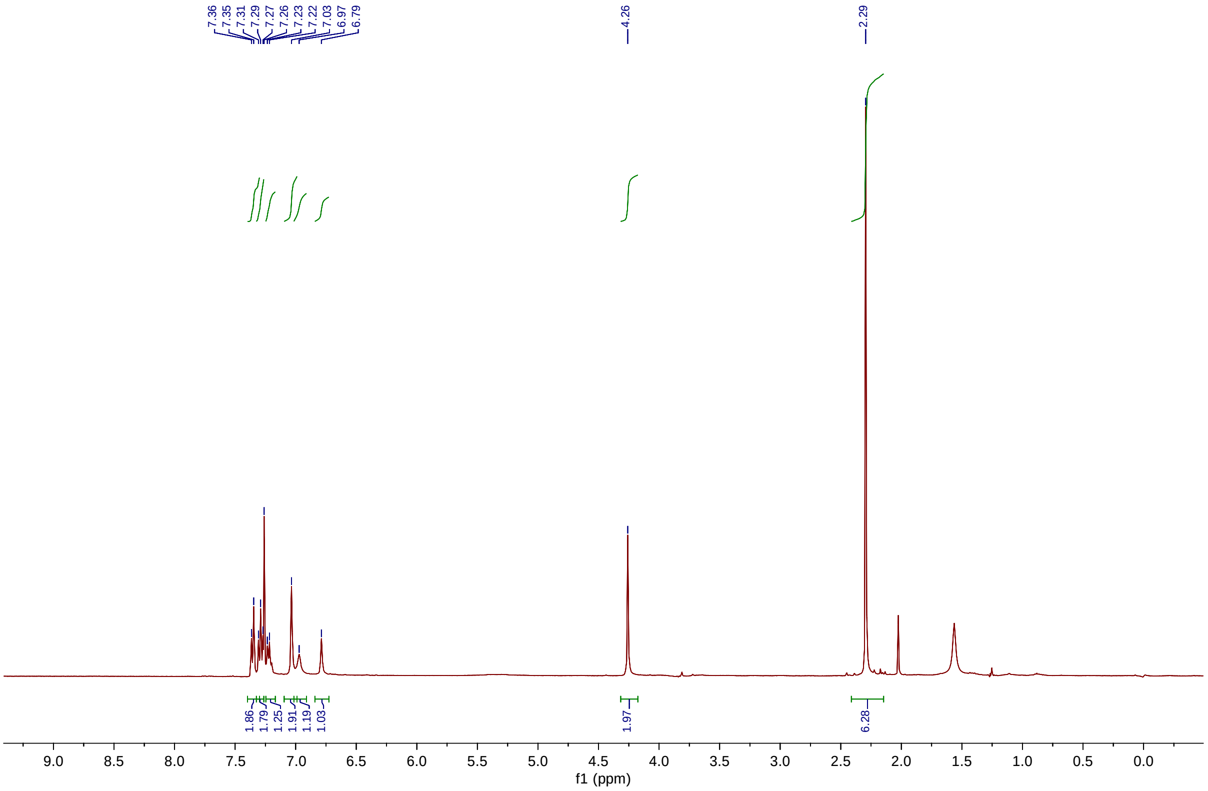

^1H NMR Spectrum of compound **3o** ($CDCl_3$, 400 MHz)

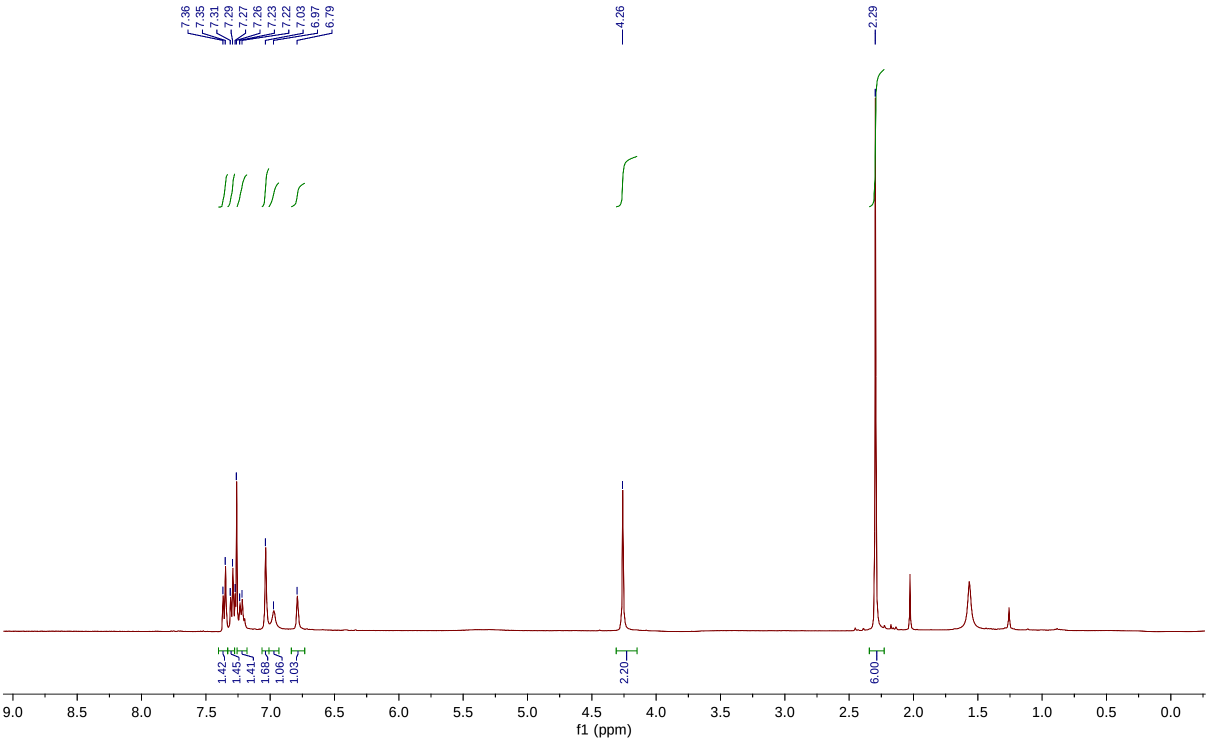

^1H NMR spectrum of the raw material recovered after stirring the selenocarbamate **3o** in the buffer solution and under conditions used for the kinetic assays.

3. Structure determination

The crystal structure of hCA II (PDB accession code: 4FIK) without solvent molecules and other heteroatoms was used to obtain initial phases using Refmac5.[2] 5% of the unique reflections were selected randomly and excluded from the refinement data set for the purpose of Rfree calculations. The initial |Fo - Fc| difference electron density maps unambiguously showed the inhibitor molecules. The inhibitor was introduced in the model with 1.0 occupancy. Refinements proceeded using normal protocols of positional, isotropic atomic displacement parameters alternating with manual building of the models using COOT.[3] The quality of the final models were assessed with COOT and RAMPAGE.[4] Atomic coordinates were deposited in the Protein Data Bank (PDB accession code: 7QBH). Graphical representations were generated with Chimera.[5]

4. Summary of Data Collection and Atomic Model Refinement Statistics for hCA II

	**hCAII + 3o**
PDB ID	7QBH
Wavelength (Å)	0.971700
Space Group	P21
Unit cell (a, b, c, α, β, γ) (Å,°)	42.38, 41.48, 72.33, 90.00, 104.53, 90.000
Limiting resolution (Å)	41.47-1.22 (1.25-1.22)
Unique reflections	58985 (1034)
Rmerge (%)	6.3 (28.1)
Rmeas (%)	6.9 (38.3)
Redundancy	5.47 (2.05)
Completeness overall (%)	81.9 (19.6)
<I/σ(I)>	15.65 (2.36)
CC (1/2)	99.8 (82.6)
**Refinement statistics**	
Resolution range (Å)	41.47-1.22
Rfactor (%)	15.23
Rfree(%)	17.70
r.m.s.d. bonds(Å)	0.0145
r.m.s.d. angles (°)	1.9515
**Ramachandran statistics** (%)	
Most favored	96.9
additionally allowed	3.1
outlier regions	0.0
**Average B factor ($Å^2$)**	
All atoms	13.754
inhibitors	22.519
solvent	22.646

5. Crystallographic figures

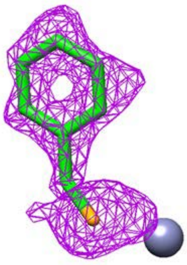

Figure S1. Electron density of inhibitors **3o** bound to zinc (grey) in hCA II active site. $2F_o$-F_c maps and contoured to the 1.0 σ level.

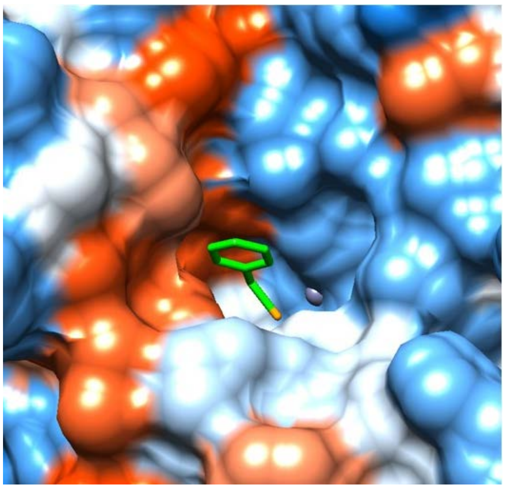

Figure S2. Compound **3o** inside the active site of hCA II. Hydrophobic (red) and hydrophilic (blue) residues are labeled

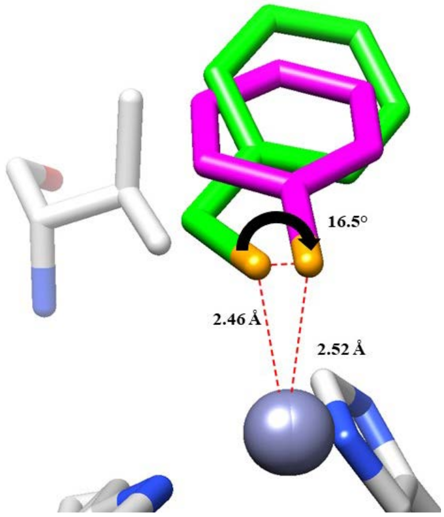

Figure S3. Overlay of compounds **3o** and **5** with hCA II

6. References

[1]. A. Capperucci, A. Petrucci, C. Faggi, D. Tanini, *Adv. Synth. Catal*. **2021**, *363*, 4256-4263.

[2]. G. N. Murshudov, A. A. Vagin, E. J. Dodson, *Acta Crystallogr. D Biol. Crystallogr*. **1997**, *53*, 240-255.

[3]. P. Emsley, B. Lohkamp, W. Scott, K. Cowtan, *Acta Crystallogr. D Biol. Crystallogr*. **2010**, *66*, 486-501.

[4]. S. C. Lovell, I. W. Davis, W. B. Arendall III, P. I. W. de Bakker, J. M. Word, M. G. Prisant, J. S. Richardson, D. C. Richardson, *Proteins*, **2003**, *50*, 437-450.

[5]. E. F. Pettersen, T. D. Goddard, C. C. Huang, G. S. Couch, D. M. Greenblatt, E. C. Meng, T. E. Ferrin, *J. Comput. Chem.* **2004**, *25*, 1605-1612.
